# An integrative review of potential enablers and barriers to accessing mental health services in Ghana

**DOI:** 10.1186/s12961-018-0382-1

**Published:** 2018-11-16

**Authors:** Eric Badu, Anthony Paul O’Brien, Rebecca Mitchell

**Affiliations:** 1 Faculty of Health and Medicine, School of Nursing and Midwifery, The University of Newcastle, Newcastle, NSW Australia; 2 Faculty Health and Medicine, School of Nursing and Midwifery, The University of Newcastle, Newcastle, 2308 NSW Australia; 3 Health Services Research Centre, Faculty of Business and Economics, The University of Newcastle, Newcastle, NSW Australia

**Keywords:** Ghana, Psychiatry, Mental health, Access, Service users, Systems, Facilitators, Barriers

## Abstract

**Introduction:**

The importance of accessible mental health treatment is a global concern, particularly when one in five people will experience a mental health problem in their lifespan. This is no less important in Ghana; however, no studies have yet attempted to appraise and synthesise the potential enablers and barriers to accessing services in Ghana. The aim of this integrative review is therefore to identify and synthesise existing evidence on the barriers and enablers to accessing mental health services in Ghana.

**Methods:**

A search of the published literature was conducted using Medline, EMBASE, PsycINFO, CINAHL (EBSCO), Web of Science, and Scopus electronic databases. The search was limited to papers published in English and within 2000–2018. Using pre-defined inclusion and exclusion criteria, two reviewers independently screened the titles and abstracts of the retrieved papers. A data extraction form and a Critical Appraisal Checklist were used to extract and appraise data, respectively. The integrative review incorporates both qualitative and quantitative data into a single synthesis.

**Results:**

Out of 42 papers that met the inclusion criteria, 50% used qualitative methods, 33.3% used mixed methods and 16.7% used quantitative methods alone. The potential barriers in accessing mental health services were attitudinal, knowledge about services, treatment cost, transportation and geographical proximity, as well as perceived efficacy of medication. Similarly, the health systems factors contributing to barriers were low priority, limited funding sources, irregular medicine supply, limited services for marginalised groups and poor state of psychiatric facilities, together with poor management of mental health cadres. The potential enablers for service users involved increased decentralisation and integration, task-shifting and existing support services.

**Conclusion:**

The existing evidence on mental health in Ghana is skewed towards weaknesses in the systems and stigma, with rationally little, or no, evidence or emphasis on the effectiveness, or quality of mental health services. These attributes largely neglect the provision of psychiatric services for marginalised mental health service user groups, including children, adolescents, people with disabilities and the elderly.

**Electronic supplementary material:**

The online version of this article (10.1186/s12961-018-0382-1) contains supplementary material, which is available to authorized users.

## Introduction

Globally, mental, neurological and substance (MNS) use disorders are estimated to affect 450 million people, causing one in every five people to experience a mental health issue at some point in their lives [1]. The global burden of MNS has increased at a rate of 37.6% over the past 20 years due to several factors, including aging and population growth [1]. The existing estimates suggest that, in 2015, 4.4% of people, representing 322 million of the global population, experienced a depressive episode, whilst 3.6% were affected by anxiety disorders [2]. The global burden of depression varies across regions, with 27% in South-East Asia, 12% in Europe and 9% in Africa [2]. In Ghana, WHO estimates indicate that approximately 2.1 million people suffer from moderate to severe mental illness, 650,000 of whom are projected to have a severe mental illness [3].

Generally, people with MNS use disorders are characterised by three major dimensions, and include a person diagnosed with non-organic psychosis, personality disorder, a more prolonged episode (approximately 2 years), or a longer history, as well as those consumers with longitudinal treatment needs [4]. WHO defines mental health as a state of well-being, in which the individual realises their own potentials, ability to cope with the normal stresses of life, functionality and work productivity as well as the ability to contribute efficiently in community life [5]. Similarly, the Ghana Mental Health Act 846 defines mental disorders as “*a condition of the mind in which there is a clinically significant disturbance of mental or behavioural functioning associated with distress, or interference of daily life and manifesting as disturbance of speech, perception, mood, thought, volition, orientation, or other cognitive functions to such a degree as to be considered pathological but excludes social deviance without personal dysfunction*” (Government of Ghana [6]). The concept of MNS across different settings is categorised into various forms, ranging from moderate to severe conditions depending on the functional capacity. Severe MNS use disorders are measured depending on the diagnosis, degree of disability and the presence of abnormal behaviour [4].

Traditionally, whilst there is an increasing global burden of MNS use disorders, there are several difficulties in delivering mental health services, particularly in low- and middle-income countries (LMICs) [7–12]. Accordingly, existing assessments suggest that only 25% of mental health service users in LMICs have access to services, which indicates a high unmet need for the availability of mental health services to match the increasing burden of mental health conditions. Mounting literature on MNS use disorders in LMICs have identified several factors that account for the high unmet needs to mental health services [7, 9]. These factors occur at the health systems and individual patient levels. In most health systems in LMICs, MNS use disorders are less prioritised, which subsequently leads to limited policy, legislation and regulation to back the operation of mental health services [7, 9]. Until recently, most mental health systems in Africa were not supported by mental health policy and regulations that reflect international research or policy recommendations. Even in settings where such documents exist, there is evidence of poor implementation and coordination [8, 9, 13]. Sometimes, there is poor consultation and a lack of evidence-based policy formulation that will translate to implementation [9]. Until now, most existing mental health policies and regulations in LMICs, including Ghana, have not involved users of mental health services in policy formulation [14]. In contrast, in developed countries, consumer involvement is now a mandatory consideration in the development and operation of mental health services.

Similarly, mental health systems in LMICs are historically challenged, with limited funding support, human resources and poor infrastructure that is responsive to the ever-increasing demand created by mental health service users. Subsequently, service users in these settings experience widespread stigma from the public [7, 9, 15–17]. The stigmatising attitudes are caused by socio-cultural and belief systems of these societies. In these settings, the public hold a high negative perception that MNS use disorders are caused by spirituality and supernatural forces. Undoubtedly, these perceptions hinder service users from seeking treatment.

Recently, an increasing number of empirical studies on mental health services in Ghana have been undertaken. These studies employ both qualitative and quantitative primary data and are largely focused on the gaps in policies, treatment pathways, mental health systems weaknesses and caregivers’ experiences. However, despite this growing literature, there has been no review that systematically appraises and synthesises evidence on the enablers and barriers in accessing mental health services in Ghana, nor on the effectiveness of the services for service users.

This paper aims to address this gap by undertaking an integrative review into issues pertaining to user and caregiver access to mental health services in Ghana. A preliminary search conducted from Medline, Web of Science, Google Scholar, Scopus Index and EMBASE identified only two reviews. One of these focused on the direction of neuroscience-related research in Ghana [18], whilst the other focused on the state of mental health research, but only on the estimation of epidemiological data on MNS use disorders [19]. Although these papers attempted to synthesise the existing evidence on mental health services, they did not address the potential enablers and barriers faced in accessing services. The lack of such evidence compromises effective system planning, monitoring and delivery of quality mental health services.

Consequently, identifying, appraising and developing an aggregated synthesis research evidence on the potential enablers and barriers to accessing mental health services is of significance to policy-makers, academia and service providers, as well as service users themselves. Undoubtedly, the evidence is expected to unveil the potential enablers and barriers to inform policy decision-making. Moreover, understanding the potential barriers confronting service users can inform stakeholders, including health professionals, about the existing needs of service users and subsequently assist to shape patient-centred service delivery. Again, the synthesis would inform stakeholders about the gaps in the literature, as well as the direction and priority areas of mental health research in Ghana.

### Aim

The aim of this integrative review is to identify, appraise and develop an aggregated synthesis of mixed method research evidence on the enablers for and barriers to accessing mental health services in Ghana.

### Objectives

1. To identify the existing enablers facilitating access to psychiatric services
2. To synthesise the existing barriers confronting mental health service users to accessing mental health services

## Methods

An integrative review with a mixed method approach was conducted to synthesise evidence on the potential enablers and barriers to accessing mental health services. The integrative review incorporated both qualitative and quantitative data as well as both experimental and non-experimental research [20, 21]. The integrative review employed a five-stage approach, which included problem identification, literature search, data evaluation, data synthesis and presentation of results [21].

### Eligibility criteria

The integrative review included papers that use quantitative, qualitative data or both. The qualitative studies comprised a wide range of studies that applied phenomenological, exploratory, ethnography and participatory action research. The qualitative element also included commentaries and expert opinions. The quantitative papers included studies that employ cross-sectional design, primary data collection using questionnaires and secondary data, or document review. The papers included in this integrative review were limited to those published in English within 2000–2018. The integrative review included papers that explore the treatment pathways, enablers and barriers to accessing mental health services. Similarly, the integrated review included papers that measures the extent at which the mental health service provision function in Ghana.

#### Participants

The integrative review included papers that target multiple participant groups, including stakeholders from government ministries, health professionals, mental health service users, family members and caregivers as well as community members.

### Exclusion criteria

The integrative review excluded studies that did not target the variables of interest (treatment pathways, enablers and barriers to accessing mental health services). Papers published before 2000 and those published in languages other than English were also excluded.

### Search strategy

This review incorporated all peer-reviewed published papers focusing on mental health services in Ghana. The search of published papers was conducted from electronic databases, including MEDLINE, EMBASE, PsycINFO, CINAHL (EBSCO), Web of Science and SCOPUS. As recommended by the Joanna Briggs Institute (JBI) guidelines for conducting systematic reviews [20], several steps were utilised to conduct the search of information. Firstly, an initial search was conducted in MEDLINE and SCOPUS, followed by analysis of the search items found in the titles and abstracts in the articles. Secondly, the keywords and index terms identified were used to search articles across all the selected databases. The final stage involved hand searching of the reference list of all identified articles for additional papers of relevance.

### Search terms and Boolean operators

“Mental disorders” or “anxiety disorders” or “mental illness” or “mental health” or “bipolar and related disorders” or “elimination disorders” or “neuro-developmental” or “neurotic disorders” or “schizophrenia spectrum” or “psychotic disorders” or “substance-related disorders” or “trauma and stressor related disorders” or “psychiatric case” or “services users” or “psychiatric patient” or “treatment barrier” or enabler* or facilitator* or barrier* or uptake* or participat* or utiliz* or utilis* or avail* or aware* or “patient participation” or Geograph* or proximity* or affordab* or physical* or locat* or availab* or accessib* or facilitator* or accept* or stigma* or discriminat* or biomed* or faith-bas* or psychosoc* or quality* or attitud* or inclus* or equit* or “service use” or uptake* or treatment*.

### Selection process

**Fig. 1:**
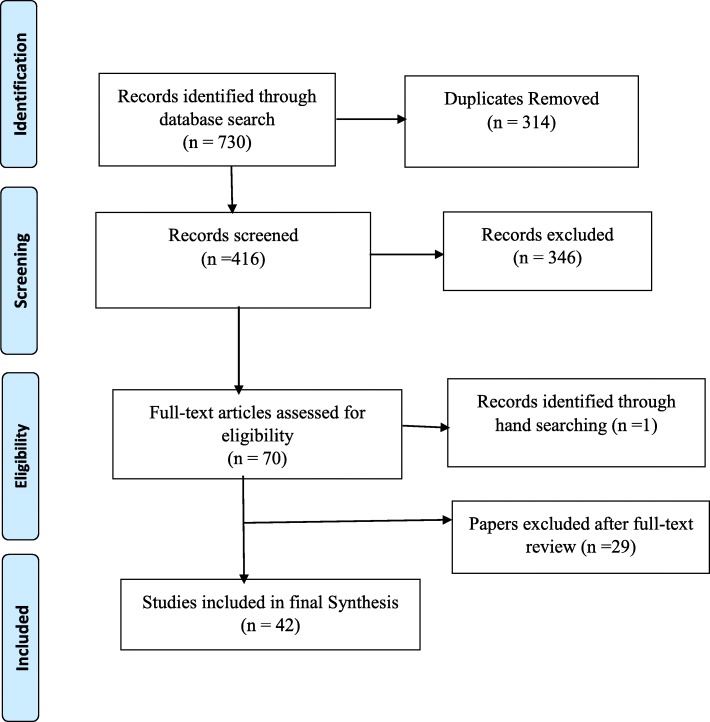
Flow chart of studies included in the review

The selection of articles was conducted through several stages. Two authors independently screened the titles of articles that were retrieved and then approved those meeting the selection criteria. Secondly, the authors reviewed all the abstracts and agreed on those needing full-text screening. Accordingly, in the full text screening, all the information and records that were approved were included in the final review. The Preferred Reporting Items for Systematic Reviews and Meta-Analyses (PRISMA) flow chart for systematic reviews [22] was used to illustrate the selection processes (Fig. 1).

### Data management and extraction

The study used Endnote X8 to manage the search results and screening, particularly handling duplicate references. Two reviewers independently managed the data extraction process. Data was extracted from all included papers using pre-defined data extraction criteria (Appendix). The data extraction form was developed using Cochrane [22] and the JBI manuals [20], as well as consultation with experts in methodologies and the subject area. The data extraction forms focus on different subsections, which include study details, methodological characteristics, policies and laws on mental health, epidemiological data on service users, potential enablers to accessing services, potential barriers to accessing services, perceived effectiveness of existing services, as well as additional information on mental health, recommendations and additional references to follow up on.

### Assessment of methodological quality/critical appraisal

Two reviewers independently appraised the methodological quality of all papers prior to their inclusion in the final review. The authors developed a critical appraisal check list using the JBI [23] and Mixed Methods Appraisal Tool [24] critical appraisal tools. The various sections that were captured in the appraisal tools were divided into six subsections, focusing on various methods and designs, including Qualitative, Quantitative randomised controlled trials, Quantitative non-randomised (analytical cross-sectional), Quantitative descriptive, Systematic review and Mixed methods. Each of the subsections had specific questions related to methodological and reporting quality (Additional file 1). The aim of this appraisal was to better understand whether to include or exclude articles or seek further information from the authors. Overall, the methodological quality score was assessed by assigning low quality (a score below 25%), medium quality (a score of 50%) and high quality (a score of 75% and above) scores. These scores were computed by counting the number of ‘Yes’ answers for each subsection of the methodological criteria and expressing them as a percentage [24].

### Data synthesis

The extracted data were analysed using a mixed methods synthesis [20]. Both quantitative and qualitative data were assimilated into a single synthesis and a content analysis process applied to derive commonalities. The qualitative data were coded and presented with the quantitative data in a meta-aggregation [20]. The coded ideas were assigned alphabetically and colour coded. The coding process was repeated for all the extracted data. These codes were further grouped into emerging subthemes, and subsequently combined as a framework. The qualitative data were converted into a numerical format and combined with the quantitative data in a descriptive analysis. The key emerging themes were categorised into a table format as an analytical framework. The background information of included papers and emerging codes were analysed using STATA version 15.

### Theoretical framework

The Penchansky and Thomas theory on access to health services was adapted to organise and discuss the findings of the integrative review [25–28]. The theory defines access as a general concept that summarises a set of more specific dimensions, describing the fit between service users and the health systems [26]. Conversely, a recent synthesis defines access as the ability to identify healthcare needs, seek care, optimum use of healthcare resources and receipt of the needed health services [28].

The initial Penchansky and Thomas theory proposed five dimensions, including Accessibility (location), Availability (supply and demand), Acceptability (consumer perception), Affordability (financial and management related cost) and Adequacy (organisation). These dimensions have heavily been criticised as weak due to the inability to cover the varied spectrum of access to health services, and therefore received several expansions. Subsequently, Saurman [25] expanded the original Penchansky and Thomas theory to six dimensions, with the addition of Awareness, whilst Russell and Humphreys [26] conceptualised the seven dimensions of access, with the addition of three additional components (Accommodation, Awareness and Timeliness). The extension of these dimensions helps to cover a broader spectrum of factors that can facilitate health service utilisation. Additionally, Levesque and Harris [28] suggested five dimensions of access to health services (Approachability, Acceptability, Availability or Accommodation, Affordability and Appropriateness), which is comparable to Penchansky and Thomas theory, though there are differing terms used to describe the concept. The dimensions used to facilitate the findings from our integrative review were Awareness, Availability, Adequacy (Accommodation), Accessibility (Approachability), Affordability and Acceptability (Appropriateness). The key important issue that needs to drive the application of the dimensions in this theory is the extent and ability to facilitate the use of mental health services. This practically translates as the ability to perceive, seek, reach, pay and engage [28].

## Results

### Description of papers retrieved

The study identified a total of 730 papers from various sources, after which 314 duplicates were removed. Out of this, 416 records were screened and 70 met the inclusion criteria, whilst 346 were excluded. After a review of the 70 full text articles and methodological quality assessment, one additional paper was identified from the reference lists and 29 papers further excluded, with 42 papers included in the final synthesis (Fig. 1). Out of the 42 included papers, 34 had high quality evaluation scores, 6 had medium and 2 had low scores (Table 1).

**Table 1:** Methodological quality ratings of included studies

Included paper	Study design	Type of study	Sampling	Type of data	Type of analysis	S^a^ (%)
Adjorlolo, Abdul-Nasiru [32]	Cross-sectional	Quantitative	Not reported	Questionnaire	Inferential statistics	90
Ae-Ngibise, Cooper [45]	Not reported	Qualitative	Purposive	IDIs and FGD	Thematic analysis	75
Ae-Ngibise, Adiibokah [42]	Longitudinal	Mixed methods	IDIs and review	Not reported	Thematic analysis	60
Agyapong, Farren [47]	Cross-sectional	Mixed methods	Questionnaire	Purposive	Descriptive and inferential statistics	73
Agyapong, Osei [56]	Cross-sectional	Mixed methods	Questionnaire	Purposive	Descriptive and inferential statistics	77
Agyapong, Osei [31]	Cross-sectional	Mixed methods	Questionnaire	Purposive	Descriptive and inferential statistics	77
Agyapong, Osei [51]	Cross-sectional	Mixed methods	Questionnaire and IDIs	Purposive	Descriptive statistics and Thematic content analysis	77
Agyapong, Osei [48]	Cross-sectional	Mixed methods	Questionnaire	Purposive	Descriptive and inferential statistics	63.6
Akpalu, Lund [55]	Cross-sectional	Mixed methods	IDIs and WHO Assessment Instrument	Purposive	Thematic analysis	73
Appiah-Poku, Laugharne [58]	Not reported	Quantitative	Questionnaire	Not reported	Not reported	81
Arias, Taylor [59]	Not reported	Qualitative	IDIs	Purposive	Constant comparative	75
Asamoah, Osafo [44]	Not reported	Qualitative	IDIs	Not reported	Thematic analysis	75
Awenva, Read [30]	Not reported	Mixed methods	IDIs, FGD and WHO Assessment Instrument	Not reported	Thematic analysis	73
Bhana, Petersen [46]	Not reported	Mixed methods	IDIs, FGD and WHO Assessment Instrument	Not reported	Thematic analysis	73
Bird, Omar [29]	Not reported	Qualitative	IDIs	Purposive	Thematic analysis	75
Canavan, Sipsma [49]	Not reported	Quantitative	Document review	Not reported	Descriptive and inferential statistics	63.6
Cohen, Raja [43]	Not reported	Qualitative	Participatory - Observations, field notes taking	Not reported	Not reported	87.5
de Menil, Osei [60]	Cross-sectional	Quantitative	Questionnaire	Random, stratified and purposive	Descriptive and inferential statistics	100
Doku, Ofori-Atta [41]	Not reported	Qualitative	IDIs and FGD	Purposive	Thematic analysis	100
Gyamfi, Hegadoren [61]	Descriptive	Qualitative	In-depth interview	Not reported	Thematic analysis	75
Ibrahim, Hor [62]	Cross-sectional	Quantitative	Questionnaire	Purposive	Descriptive and inferential statistics	90.9
Jack, Canavan [36]	Not reported	Qualitative	IDIs	Snowballing	Constant comparative	75
Kleintjes, Lund [10]	Not reported	Mixed methods	IDIs, FGD and WHO Assessment Instrument	Purposive	Thematic analysis	60
Kpobi and Swartz [63]	Not reported	Qualitative	IDIs	Snowballing	Thematic analysis	62.5
Kpobi and Swartz [52]	Not reported	Qualitative	IDIs	Snowballing	Thematic analysis	75
Kyei, Dueck [64]	Not reported	Mixed methods	Questionnaire	Random sample	Descriptive and inferential statistics	63.6
Mfoafo-M’Carthy, Sottie [37]	Not reported	Qualitative	Document review	Not reported	Thematic analysis	45
Ofori-Atta, Attafuah [53]	RCT	Quantitative	Questionnaire	Randomisation	Descriptive and inferential statistics	100
Ofori-Atta, Attafuah [35]	Not reported	Mixed methods	IDIs and WHO Assessment Instrument	Purposive	Thematic analysis	80
Opare-Henaku and Utsey [38]	Not reported	Qualitative	IDIs	Not reported	Thematic analysis	100
Oppong, Kretchy [65]	Not reported	Qualitative	IDIs	Convenience	Thematic analysis	100
Osafo [66]	Not reported	Qualitative	Review	Not reported	Synthesis	20
Osafo, Agyapong [67]	Not reported	Qualitative	IDIs	Purposive and Snowballing	Thematic analysis	75
Quinn [33]	Not reported	Qualitative	IDIs	Not reported	Descriptive	75
Raja, Wood [50]	Not reported	Qualitative	IDIs and document review	Purposive	Descriptive statistics and Thematic content analysis	100
Read [57]	Ethnographic	Qualitative	IDIs, FGD and observation	Purposive	Grounded theory approach	75
Read, Adiibokah [54]	Ethnographic	Qualitative	IDIs and FGD	Not reported	Not reported	100
Roberts, Mogan [40]	Not reported	Mixed methods	WHO-AIMS tool and Questionnaire	Purposive	Thematic analysis	86.6
Salifu Yendork, Kpobi [39]	Phenomenological	Qualitative	IDIs, FGD and observation	Purposive, Snowballing and Convenience	Interpretative Phenomenological analyses	100
Stefanovics, He [34]	Exploratory	Qualitative	Questionnaire	Convenience	Descriptive and inferential statistics	72.7
Tawiah, Adongo [68]	Cross-sectional	Mixed methods	IDIs and FGD	Random sample	Descriptive and inferential statistics	77
van der Watt, Nortje [69]	Not reported	Qualitative	FGD	Not reported	Thematic analysis	100

*FGD* focus group discussion, *IDI* in-depth interview

^a^Methodological quality score

### Study characteristics

Overall, 42 papers were included in the integrative synthesis. Most papers (85.7%) were of studies conducted in Ghana alone, whilst 14.28% were multi-country studies that included Ghana. Out of the included papers, 59.52% did not report the study design, 23.81% used a cross-sectional design and 2.47% used an ethnography design. Five in every 10 (50%) of the included papers used qualitative methods, 33.33% used mixed methods (both quantitative and qualitative) and 16.67% used quantitative methods alone. Similarly, more than half (54.76%) of the included papers used non-probability sampling (purposive, snowballing and convenience), whilst 9.25% used probability sampling (simple random and stratified sampling).

Additionally, more than half (54.76%) of the included papers analysed the studies using various approaches (constant comparative, thematic content and interpretative phenomenological analysis) whilst approximately one-third (30.95%) used descriptive and inferential statistics. The majority of the papers (76.19%) involved multi-stakeholder groups as participants (policy-makers, psychiatrics, mental health nurses, users of psychiatric services, teachers, police officers, academics, herbalists, Islamic healers, Christian faith healers and traditional shrine), whilst only 14.28% involved users of psychiatric services alone. Again, more than one-third (40.47%) of the included papers measured mental health systems, 38.09% measured treatment pathways and 21.42% measured attitudes towards mental illness (Table 2).

**Table 2:** Background information of included papers

Variable	Frequency	Percentage
Study design		
Not reported	25	59.52
Cross-sectional design	10	23.81
Descriptive	1	2.38
Longitudinal	1	2.38
Exploratory	1	2.38
Phenomenological	1	2.38
Ethnography	2	4.76
Randomised controlled trial	1	2.38
Type of study		
Mixed methods	14	33.33
Qualitative	21	50.00
Quantitative	7	16.67
Sampling		
Probability	4	9.25
Non-probability	23	54.76
Not reported	15	35.71
Type of analysis performed		
Qualitative analysis^a^	23	54.76
Descriptive and inferential	13	30.95
Descriptive statistics and content	2	4.76
Not reported	3	7.14
Outcome of included papers		
Treatment pathways	16	38.09
Mental health systems	17	40.47
Attitudes towards mental illness	9	21.42
Type of participants		
Multi-stakeholder groups	32	76.19
Mental ill patients	6	14.28
Community members	3	7.14
Study setting		
Multi-country	6	14.28
Ghana alone	36	85.71

^a^Constant comparative, thematic and interpretative phenomenological analysis

### Awareness

The concept of awareness indicates the use of effective communication and information strategies to make mental health services known to relevant stakeholders, including service users, providers and the broader community [25]. Subsequently, applying this concept can be effective if there is adequate health literacy, particularly the knowledge and belief about mental illness [28].

#### Barriers to access related to Awareness

##### Low priority

Some studies put forward that mental health services have received low priority at both national and regional levels, compared with other competing health and developmental programmes [10, 29, 30] (Table 3). Additionally, Bird and Omar [29] showed that mental health issues are explicitly excluded from national development priorities.

**Table 3:** Key themes of potential enablers and barriers

Theory	Themes	Subthemes	Number of studies	Papers
Awareness	Barriers to access related to awareness	Knowledge about psychiatric care	2	[29, 31]
		Attitudes towards service users	12	[29, 31–39, 42, 64]
		Low priority	3	[10, 29, 30]
	Enablers to access related to awareness	Support services	9	[29, 33, 39, 40, 42–44, 46]
		Mental health law	3	[30, 40, 41]
Affordability	Barriers to access related to affordability	Treatment and medication cost	6	[31, 33, 35, 49, 50, 52]
		Limited funding	6	[29, 30, 35, 40, 46, 50]
Accessibility	Barriers to access related accessibility	Transportation and geographical proximity	2	[40, 54]
	Enablers to access related to accessibility	Decentralisation and integration	4	[40–42, 55]
		Referral system	4	[35, 40, 51, 56]
		Irregular medicine supply and prescription	6	[30, 35, 40, 46, 49, 50]
		Poor state of psychiatric facilities	4	[30, 31, 35, 36]
		Poor management of mental health cadres	8	[30, 31, 35, 36, 40, 46–48]
	Enablers to access related to accessibility	Task-shifting		[35, 40, 42, 47, 51]
Acceptability	Barriers to access related acceptability	Perceived efficacy of medication	7	[30, 36, 47, 49, 54, 56, 57]
		Relapsed treatment	3	[49, 55, 57]
		Discontinuity of medications	1	[57]
Adequacy	Barriers to access related to adequacy	Limited mental health services for marginalised groups	3	[35, 40, 46]

##### Knowledge about psychiatric care

Some studies reported that service users lack knowledge about the appropriate interventions to prevent and promote mental health, as well as where to obtain biomedical treatment [29, 31]. Accordingly, the lack of relevant information on existing mental health services could hinder service users from seeking appropriate interventions.

##### Attitudes towards service users

Most studies described several attitudes exhibited against service users (Table 3). These attitudes were categorised as positive or negative [32, 33], sympathetic or unsympathetic [32], favouring [34] and stigmatising [31, 35, 36]. Stigma largely originates from the public, family members and service users themselves, and sometimes influences family members to abandon service users at psychiatric hospitals [35, 36]. Two papers explained that the media, which appears as a significant source to change public perception and reduce stigmatising attitudes, used several derogatory terms and remarks to describe mental health service users. Such reporting also suggested to be an exemplar of public opinion being stigmatised toward those people living with mental illness. The stigmatising attitudes literally portrayed people with mental illness as unpredictable, violent, dangerous [37, 38], displaying unintelligible speech, talking to themselves or with an unkempt physical appearance, with these symptoms argued to pose a higher level of risk to themselves and others [37–39]. These stigmatising attitudes not only perceive people with mental illness as dangerous but prevent them from seeking biomedical treatment at the psychiatric facility. The stigmatising attitudes are not only limited to mental health service users and their families, but also associated with those who worked in mental health specialties, including health workers and policy-makers [29, 31].

#### Enablers to access related to Awareness

##### Mental health law

Some studies demonstrated that the Ghana mental health law (Act 846) of 2012 presents a new era of improving the well-being of people with mental illness [30, 40, 41] (Table 3). The new law has the potential and mandate to improve the quality of mental healthcare and subsequently protect the human rights of service users [30, 41], and was developed with extensive consultation with stakeholder groups, including mental health professionals, care givers and community members [30, 41]. The increased participation of these stakeholder groups has the ability to capture the needs of people with mental illness into the law.

##### Supporting services

The integrative review highlighted that there are existing support services that could facilitate access to mental health services for service users [29, 33, 36, 39, 40, 42–45]. Generally, the support services were provided through formal and informal sources. Subsequently, the formal support services are provided through self-help groups, which include caregivers, service users, NGOs and professional associations [29, 40, 42, 43]. For instance, until 2011, there were a total of eight NGOs or consumer associations working on mental health issues in Ghana (Ghana Mental Health Association, Mindfreedom, Alcoholics Anonymous, The Epilepsy Association, Basic Needs, World Vision, Psychomental Health International). Subsequently, one of these NGOs (Basic Needs) has supported the formation of self-help groups for people with mental illness in the three northern regions of Ghana. Such self-help groups have been demonstrated as effective in diverse ways, particularly changing the help-seeking behaviour of service users towards biomedical treatment, providing financial support for treatment, supporting service users to collect medications, counselling members and providing mental health education [43]. Sometimes, the self-help groups demonstrated the potential to re-integrate individuals into communities, increase family acceptance of people with mental illness, and reduce overprotection of children with mental illness by families and caregivers [43].

In addition, another community-based mental health project in the Kintampo district of Ghana supported the formation of self-help groups composed of people with mental illness and caregivers, and subsequently helped them employ a social determinants approach to managing mental illness [42]. Again, the support services provided by mental health professionals have the potential to facilitate access to mental health services. In some instances, mental health workers demonstrated an increasing desire to change stigmatising attitudes towards mental health, particularly correcting the misunderstandings that mental illness is treatable and increasing respect for the field of mental healthcare [36].

Alternatively, one paper described that mental health service users, families and carers received informal support services, such as emotional support, from different sources, which included extended family members, local community, friends, church and mosque [33]. Additionally, some studies highlighted that churches provide several support services, which include mental health education [39, 44, 45]. The mental health education provided by churches usually focused on the causes, prevention, caring for, and reducing stigmatising and discrimination as well as life enhancement. These social support services include financial support, emotional care, and informal and unprofessional counselling. These services are perceived to be comparable to psychosocial services provided by ‘clinical psychologists’ [45].

### Availability

Availability explains the capacity and strength of the existing mental health services, including facility characteristics, densities, concentrations and distribution [28]. Subsequently, applying Availability suggests the ability to make a socially and culturally accepted mental health service easily available to service users.

#### Barriers to access related Availability

##### Poor state of psychiatric facilitates

The integrative review highlights that the existing state psychiatric hospitals face several challenges [30, 31, 35, 36]. These challenges are experienced in areas such as overcrowded facilities, poor state of infrastructure, as well as limited tools and equipment, including basic medical supplies and computers for keeping patient records [31, 36]. Subsequently, Ofori-Atta and Read [35] further added that the overcrowding of the psychiatric facilities is mainly caused by the lengthy stay of service users, particularly offenders who have been ordered by the courts to be admitted for a psychiatric assessment as well as those with limited financial support.

##### Poor management of mental health cadres

The integrative review demonstrates that there is poor management of human resources for mental health services in Ghana [30, 31, 35, 36, 40, 46–48]. Some studies indicated that the current strength of mental health professionals is limited to meet the increasing demand of mental health services [30, 35, 36, 40, 46, 47]. For instance, a sample of 29 health policy directors (89.7%) as well as 11 psychiatrist staff perceived that the number of mental health professionals in Ghana is completely inadequate [47]. Another paper attributed this challenge to an aging workforce and increasing migration of specialists outside the country or to other specialisations [46].

Additionally, there are several gaps that hinder the existing mental health professionals’ delivery of mental health services. Although the training of community mental health workers (CMHWs), including community mental health officers (CMHOs), clinical psychiatric officers (CPOs) and community psychiatric nurses (CPNs) is relevant to provide mental health services at the primary healthcare (PHC) level, there are gaps in the training provided to these professional groups. For instance, two papers showed that, whilst the current training of CMHWs may be adequate in relation to performing their officially assigned duties, it is inadequate in preparing them to effectively deliver the mental health roles [47, 48]. This is attributed to the fact that the training of CMHWs has some weaknesses, characterised by limited practice, of short duration and shallow in content (e.g. excludes prescribing and treatment for CMHOs), as well as limited in-service training in psychotropic medication, assessment and diagnosing [47, 48]. Similarly, the CMHWs perceived that the gaps in training are experienced in areas such as managing mental health of specific marginalised populations (including children and adolescents, elderly, pregnant women and forensic or prison population), neurological disorders (especially the management of epilepsy), risk assessment and management, medico-legal issues in psychiatry, training in psycho-social interventions, use of seclusion and physical restrain techniques as well as other tasks including report writing, team working, research methods and general administrative procedures [48].

Aside from the gaps in training, there is inadequate supervision provided to mental health professionals delivering mental health services at the PHC level [36, 47, 48]. Subsequently, one study reviewing clinical supervision and training showed that there was inadequate supervision provided to CMHWs to meet their expectations [48]. For instance, whilst 35.1% of CMHOs, 21.1% of CPOs and 9.9% of CPNs perceived that they hardly received supervision, 50% of all CMHWs perceived that there is either no supervision at all or, at most, less than once monthly supervisions [48].

Additionally, two papers perceived that mental health professionals are at higher risk of managing the aggressive behaviour of mental health service users. For instance, whilst Agyapong and Osei [31] suggested that CMHWs are affected by the risk of working in mental health, Jack and Canavan [36] concluded that these workers are unable to defend themselves from these aggressions. However, despite the increasing fear of attacks from service users and subsequent frustration, there is no compensation for risk [36].

The weaknesses in managing human resources for mental health, including an inadequate supply of mental health professionals as well as poor management of the few existing cadres, have significant implications on the provision of adequate mental health services that meet the standard of service users [30]. Mental health professionals, including psychiatric and CMHWs, have increasingly expressed their desire to quit this area of specialty. For instance, whilst 37.2% of CMHWs perceived that they have considered leaving the mental health profession [31], 63.6% of psychiatrists and 37.9% of health policy directors perceived to know CMHWs who have considered leaving the mental health profession [31]. The poor management of mental health cadres, coupled with stigmatising attitudes as well as the risk of working with people with mental illness, account for the increasing threat of leaving this area of specialty.

##### Irregular medicine supply and prescription

Most studies indicated that there is an irregular supply of medicines to meet the needs of mental health service users [30, 35, 40, 46, 49, 50] (Table 3). Although there is a list of essential medications, including antipsychotics, anxiolytics, antidepressants, mood stabilisers and antiepileptic drugs, there seems to be a shortage. In the absence of medication at the hospital pharmacies and clinics, service users are required to purchase the necessary medication from private pharmacists at their own expense, often beyond the reach of consumers, which contributes to the revolving door of non-adherence and readmission [35, 46]. Several factors were described to cause shortages of psychotropic medications, including limited government funding as well as multi-layered and unnecessarily complex bureaucratic procurement of medications [49]. Canavan and Sipsma [49] further suggested that the entire procurement process is bureaucratic and could translate to delays in drug delivery of as long as 2 years.

Another challenge that the review identified was the inconsistency in prescribing drugs to service users in the few psychiatric facilities [49]. For instance, in a sample of 7296 service users diagnosed with schizophrenia or delusional disorders, 24.1% were prescribed with anti-psychotic medications alone, whilst an additional 18.7% were prescribed with both anti-psychotic and anti-cholinergic medications. However, 8.3% of patients were prescribed no medication, although they had been diagnosed with schizophrenia or delusional disorder. Similarly, 49.0% of these service users were prescribed additional medications that were not indicated by the diagnosis listed on the encounter form or in medical records. Subsequently, whilst 83.1% of these service users had all their medications available as prescribed at the outpatient units, 11.5% could not obtain their medication through the psychiatric hospital, but had the option to purchase such medications at a private pharmacy.

#### Enablers to access related to Availability

##### Task-shifting

The integrative review showed that the Government of Ghana is currently using a task-shifting policy to increase the number of mental health professionals required to facilitate the delivery of mental health [35, 40, 42, 47, 51]. Subsequently, two new programmes, including a Community Medicine and Clinical Psychiatry (CPOs) and Diploma in Community Mental Health (CMHOs), were introduced in 2011 [40, 47]. Additionally, whilst the CPO professionals are trained to work independently in facilities without a psychiatrist, the CMHOs aimed to produce frontline community-level mental health workers to assist CPNs [40].

The task-shifting approach is recognised as effective for several reasons. This approach not only reduces the workload on the few psychiatrists but also makes mental health services easily available to service users in rural settings, subsequently reducing the problem of geographical proximity to treatment [47]. For instance, a piloted Mental Health Project in the Kintampo District from 2004 to 2010 showed that task-shifting is increasingly effective to improve access to mental health treatment for service users. The piloted intervention trained general health workers and CPNs to conduct periodic community outreach programmes, assess and manage mental illness, and facilitated access to mental health treatment for service users, subsequently facilitating collaboration with traditional healers [42].

Moreover, whilst the task-shifting strategy seems effective to facilitate access to mental health services at the primary level, there are several challenges that could impede its effectiveness. For instance, there are some exemptions and gaps in the roles assigned to mental health professionals, particularly those at the primary level. Subsequently, whilst the Ghanaian mental health system allows PHC doctors, medical assistants and community psychiatric nurses to prescribe psychotropic medications under any circumstance, PHC nurse and other workers are not allowed to prescribe psychotropic medications under any circumstance [40].

This could possibly impede effective application of task-shifting at this level. Alternatively, despite these challenges, three papers showed that there are clear assessments and treatment protocols available in the form of a standard treatment guidelines booklet [35, 40, 51]. These assessment and treatment protocols could help the service providers to provide mental health services to service users. However, these treatment protocols are not uniform in coverage to all the existing physician- and non-physician-based PHC settings. For instance, existing estimates suggest that only 1–20% of the physician- and non-physician-based PHC services had access to assessment and treatment protocols in 2011 [40].

Additionally, one key challenge in applying these assessment and treatment protocols is the gaps in knowledge of mental health professionals. For example, one paper indicated that there are variations in the knowledge of mental health professionals about the existing assessment and treatment protocols. Subsequently, whilst 83.8% of CMHOs, 94.7% of CPOs and 91.5% of CPNs from a sample had knowledge about the existing treatment protocols and could therefore guide their work, the remaining were not exposed to such treatment protocols [51].

Similarly, some stakeholders perceived that there is resistance from some mental health workers as well as traditional and spiritual healers towards the implementation of the task-shifting within mental health service delivery [47].

### Affordability

Affordability demonstrates the ability of mental health service users to afford the cost of psychiatric treatment and medication [25, 26, 28]. Alternatively, the concept describes the capacity of the mental health systems to generate resources through various financing sources to finance the cost of mental health services to service users.

#### Barriers to access related to Affordability

##### Treatment and medication cost

Some studies suggested that service users experience some challenges financing the cost of medication [31, 33, 35, 49, 50, 52]. For instance, whilst the cost of mental health treatment is subsidised for service users [49, 50, 53], they are expected to purchase their own medication from a private pharmacist whenever there is shortage of such drugs at the psychiatric facilities. Subsequently, this appears to frequently occur to service users, and so presents high costs of medication and burden [35, 49]. Additionally, Ofori-Atta and Read [35] suggested that mental health services are not covered by the National Health Insurance Scheme, and thus service users who are not insured are expected to pay for treatment for co-morbid physical conditions. The inconsistency in the pre-payment policy for service users seems to present a huge financial burden as there is loss in earnings of both the service user and the carer [35, 49].

##### Limited funding

Six papers showed that there is limited funding support for mental health services [29, 30, 35, 40, 46, 50] (Table 3). Historically, mental health services have been funded through three main sources, including government, international development partners and internally generated funds [40, 50]. Subsequently, the government funding support is provided as ring-fenced or restricted funds allocated to three psychiatric hospitals for mental health services, and used for overhead costs, including basic medical supplies and service maintenance. Raja and Wood [50] added that this funding fluctuates from year to year and therefore hospitals sometimes supplement medicine shortages from out of their operating costs [50]. Some studies indicated that there is no dedicated budget at the regional and district level to support mental health although there are existing Budget Management Centres for general health services. This problem makes it practically difficult to plan and deliver mental health services at the primary level [30, 35, 46]. Subsequently, the lack of dedicated budget support for mental health services at the district level compromised the effective integration of services into the primary health service provision [46].

### Accessibility

The concept of Accessibility suggests the ability to make services readily accessible to service users by considering the proximity in terms of time and distance [25, 26].

#### Barriers to access related Accessibility

##### Transportation and geographical proximity

Two papers showed that acknowledging the limited inpatient and outpatient mental health services at the regional and district levels has contributed to geographical access barriers (Table 3). Consequently, families and service users travel long distances to seek mental health services [40, 54]. Additionally, there is a lack of transportation for service users to access psychiatric facilities, thus presenting a geographic access barrier to mental health services [54].

#### Enablers to access related to Accessibility

##### Decentralisation and integration

The study highlighted that one of the approaches used to improve access to mental health services at regional and district levels are decentralisation and integration of mental health into general health services and community-based interventions. Subsequently, there are several psychiatric units established at general hospitals, community psychiatric nursing services at district health facilities as well as integrating mental health services into all existing regional and district health management teams. In addition, there are several initiatives from NGOs and other agencies to enhance the community detection of mental illness and provide treatment [41, 42]. For instance, a piloted mental health project in 2010 in the Kintampo District employed a community-based approach and trained general health workers to recognise and manage mental disorders [42]. The early recognition of symptoms and the establishment of therapeutic communities could help integrate patients into the community [55].

##### Referral system

Some studies showed that there are existing referral systems that could facilitate the delivery of mental health services [35, 40, 51, 56]. Two studies estimated that between 21% and 50% of non-physician PHC providers refer patients presenting with mental disorders to a higher level of care, such as the psychiatric units of the regional hospitals, or the psychiatric hospitals for at least once a month [35, 40]. For instance, approximately 79.2% of CMHWs in a previous study perceived that it was easy referring mental health service users from PHC to a psychiatric hospital [56]. This existing referral system demonstrates the extent to which the various mental health professionals work with each other at different levels. Moreover, some of the mental health workers work closely with each other at the same level to facilitate the delivery of services [51]. Subsequently, 23.8% of CMHWs were perceived to work closely with psychiatrists, 39% worked closely with social workers, 28% worked closely with psychologists and 7.9% worked closely with occupational therapists [51].

### Adequacy/Accommodation

The concept of Adequacy explains the ability to make existing mental health services accommodative or well organised to become friendly to the service user [26]. The ability to organise mental health services with the necessary provision is relevant to becoming friendly to all user groups.

#### Barriers to access related to Adequacy/Accommodation

##### Limited mental health services for marginalised groups

Three papers argue that there are limited specialist mental health services reserved for vulnerable populations including children, adolescents, elderly, people with disabilities, as well as forensic psychiatry and substance misusers [35, 40, 46]. Consequently, there are no dedicated outpatient facilities for child and adolescent mental health [35]. Additionally, Roberts and Mogan [40] suggested that, whilst there are limited community-based residential facilities for rehabilitation, the few existing facilities encountered long stays by service users.

### Acceptability/Appropriateness

Acceptability explains the perception that service users hold regarding mental health services [25]. Alternatively, the concept demonstrates the linkages between mental health services and the needs of service users, as well as the technical and perceived quality of such services.

#### Barriers to access related to Acceptability/Appropriateness

##### Perceived efficacy of medication

Most studies described the perceived efficacy of mental health services provided to service users [30, 36, 47, 49, 54, 56, 57]. Broadly, two papers suggested that, whilst psychiatric medication is recognised to be powerful and effective in the short term, particularly with the sedating effect on aggressive behaviour, there is little evidence on its effectiveness and efficacy in treating mental illness [54, 57]. Subsequently, doubts about its efficacy are attributed to the inability to achieve a permanent cure, the frequent relapse of such conditions whenever there is discontinued medication, as well as the associated unpleasant side effects [54, 57]. Additionally, five papers further described potential factors that could contribute to the perceived poor psychiatric medication outcomes [30, 36, 47, 49, 56].

Some papers indicated that there are several cases of relapse among service users (Table 3). For instance, approximately 75% and 90% of service users at the Accra Psychiatric Hospital in Ghana are those who have been treated and then relapsed, and returned unwell for readmission [55]. Several factors were identified to account for increasing cases of relapse, including non-availability of medications, lack of insight, infrequent reviews by clinicians, switching of medications [49], lack of family support, as well as lack of transport to hospitals to collect drugs [49, 57]. In addition, whilst some service users stop medication once they enter their lucid period, others discontinue antipsychotic treatment due to its unpleasant side effects including feelings of weakness and prolonged drowsiness [57].

## Discussion

### Summary of evidence

The evidence showed that the potential barriers to accessing mental health services are limited knowledge about existing services, attitudes towards service users, low priority, limited funding, irregular medicine supply, perceived efficacy of medication, limited mental health services for marginalised groups, poor state of psychiatric facilities and poor management of mental health cadres. Alternatively, some factors that equally help to facilitate access to mental health services for service users were support services, the mental health law, increasing decentralisation and integration, task-shifting and the existing referrals systems.

### Limitation

This integrative review has several limitations largely pertinent to the search words, language limitations and scope as well as the period of included papers. Firstly, the integrative review was only limited to studies conducted in Ghana. Generally, the variation in key terms and concepts regarding mental health issues may have possibly missed some relevant articles pertinent to the study. Similarly, limiting studies to only those in English and published between the years 2000 and 2018 could have missed useful articles published in other languages as well as those published prior to 2000. However, the combination of clearly articulated search methods, consultation with a research librarian, and reviewing articles with multiple experts, as well as the critical appraisal tool used to measure the methodological quality, helped to address the various limitations.

## Conclusion

Herein, we performed an integrative review on the enablers and barriers to accessing mental health services in Ghana. Of 42 included papers, 34 met the criteria for high quality and therefore had the potential to inform mental health policy and service provision. The themes identified were consistent within the dimensions of the Penchansky and Thomas’ Theory on Access [25–28], namely Awareness, Availability, Adequacy (accommodation), Accessibility (approachability), Affordability and Acceptability (appropriateness).

### Awareness

The evidence confirms that service users lack knowledge about the appropriate interventions to prevent and promote mental health as well as the place to seek psychiatric services [29, 31]. This could be ascribed to several factors, including limited advocacy and media campaigns as well as the extent of involvement of service users in the planning and delivering of psychiatric interventions. As a common characteristic in LMICs [7, 9], including Ghana, mental health services are less prioritised at several levels of the health systems. Subsequently, service users and caregivers are less involved in developing policy as well as service provision. This could possibly contribute to the limited awareness about the existing psychiatric services. In Ethiopia, for instance, whilst there tends to be wide consultation with stakeholders to develop a national strategic plan and policy, there is poor consultation in the planning as well as community and service users engagement in its implementation [9].

However, despite this limited awareness, the evidence suggests that formal and informal support services, which include self-help groups and community-based interventions, has the ability to inform service users about the existing mental health services [29, 40, 42, 43]. In fact, the formation of self-help groups from some piloted programmes in Ghana have been demonstrated to be effective in creating awareness about the existing mental health services as well as changing the help-seeking behaviour of service users [43]. Although some informal support services, such as mental health education and counselling provided by faith-healing practitioners, family and caregivers, appears unprofessional in nature, it has the ability to expose service users to the existing mental health services. Our finding implies that strengthening self-help groups as well as coordinating the activities of informal support services could likely help create awareness of the existing mental health services.

### Availability

Our findings established that the existing psychiatric facilities appear overcrowded, with poor infrastructure, basic medical supplies, long-stays by forensic patients as well as poor record keeping [30, 31, 35, 36]. Similarly, the strength of mental health professionals is limited to meeting the increasing demand of services [30, 31, 35, 36, 40, 46–48]. The limited number of mental health professionals is attributed to several factors, mostly linked to poor policy formulation and implementation, an increasing aging workforce as well as migration of specialists outside the country or to other specialisations [46]. Alternatively, among the existing professionals, there are several gaps that hinder their effective delivery of psychiatric services, which include training [47, 48], inadequate supervision as well as the risk of managing aggressive behaviour [36, 47, 48]. The weaknesses can compromise effective mental health services to meet the standard of service users. This underscores the need to address human resources in mental health, particularly their training and the effective management of their services.

Additionally, the Ghanaian mental health system is challenged with the irregular supply of medicines to meet the needs of service users and therefore presents frequent or seasonal shortages [30, 35, 40, 46, 49, 50]. Consequently, the irregular supply of medication is largely caused by health systems weaknesses, including complex and bureaucratic procurement of medicines as well as limited government funds. The lack of such medications subsequently creates barriers for service users who are already seeking in-patient mental health services and those wanting to initiate care. These constraints not only hinder the provision of quality psychiatric services but significantly prevent service users from receiving the required psychiatric services, particularly at the primary mental health level. This challenge is comparable to the functioning and characteristics of mental health systems in other LMICs [7–12]. Such constraints across most LMICs, including Ghana, are ascribed to several health systems weaknesses, including poor policy formulation and implementation as well as competing priority of mental health to other health and development issues [29, 30, 41].

However, despite these constraints, our findings identified task-shifting [35, 40, 42, 47, 51] as an effective strategy to supplement the gaps in mental health professionals. The task-shifting strategy aimed to train CMHWs, including CPOs, CMHOs and CPNs as well as general nurses to provide psychiatric services at the PHC level [40, 47]. The task-shifting not only reduced the workload on the few psychiatrists but made mental health services easily available to service users in rural settings and thus reduced the problem of geographical proximity to treatment.

### Affordability

The evidence showed that mental health services are funded through government, international development partners and internally generated sources. However, the funding is limited and unable to match the needs of the systems [29, 30, 35, 40, 46, 50]. Government funding support is provided as a ring-fenced budget allocated only to psychiatric hospitals and practically used to support the overhead costs, including basic medical supplies and service maintenance.

The health system has no dedicated budget at the regional and district level to support mental health services and thus makes it difficult to plan and deliver mental healthcare at the primary level. In fact, this challenge makes it difficult to practically integrate mental health services into the general health service. The evidence is comparable to similar characteristics of mental health systems in LMICs [7, 9].

We found that, whilst treatment and medication costs for mental health services are free, service users frequently encounter high costs of purchasing medications from private pharmaceutical providers, particularly when there are shortages of such drugs from hospital pharmacy [49, 50, 53]. Subsequently, the frequent shortage of these medications and service users being forced to purchase at their own cost present barriers in seeking mental health services [35, 49]. Our finding confirmed that, whilst mental health services are free for service users, they are absolutely not exempt from paying for the National Health Insurance Scheme, and therefore mental health service users who have no pre-payment policy tend to experience huge financial burden for co-morbid physical conditions [35, 49].

### Accessibility/Approachability

Our findings confirm that acknowledging the limited psychiatric facilities at the regional and district levels tends to present barriers to mental health service utilisation due to geographical proximity. In fact, whilst caregivers and service users, particularly those in rural communities, have to travel for long distances to seek mental health, there are limited transport services to support them. Subsequently, the long travel distance and lack of transport to support these families create geographical barriers to mental health service utilisation for service users. This finding confirms the similar trend of transportation- and geographical proximity-related barriers in other LMICs [7, 9]. Accordingly, these constraints to accessing mental health services constitute one of the major contributing factors to the high unmet needs of mental health services for service users in Ghana.

However, despite these constraints, the evidence identified decentralisation and integration [41, 42] as well as referral systems [35, 40, 51, 56] as potential enablers to facilitate the delivery of mental health services. To illustrate decentralisation and integration, community-based psychiatric units have been established at general hospitals, community psychiatric nursing services and outreach facilities at district levels. These services are practically employed to facilitate access to psychiatric services for service users at the rural setting.

### Adequacy/Accommodation

The evidence demonstrated that the Ghanaian mental health service has no specialist services reserved for vulnerable populations including children, adolescents, elderly, people with disabilities as well as forensic and substance misusers [35, 40, 46]. The lack of specialist services for these user groups has several implications, particularly regarding seeking adequate mental health services. These user groups are unlikely to receive adequate psychiatric services.

Our evidence suggest that these user groups have the opportunity to utilise the existing in-patient and outpatient psychiatric facilities commonly used by other user groups. Accordingly, recognising that these user groups require several accommodation provisions demonstrates that they are unlikely to meet their specific needs when accessing mental health services. The lack of special provisions for these user groups is largely attributed to low priority, which subsequently leads to limited recognition in policy formulation and implementation. Until recently, previous mental health laws and policies scarcely captured the needs of marginalised user groups including children, adolescents, elderly and people with disabilities [8, 10]. The limited recognition in policy could obviously account for limited provision at the service delivery level.

### Acceptability/Appropriateness

The evidence showed that psychiatric medication is recognised as effective in the short run; however, there is little evidence on its efficacy and effectiveness to permanently cure mental health conditions [54, 57]. Several factors, including service user- and system-related issues, were identified as contributing to the perceived ineffectiveness of psychiatric medication. For instance, the service user-related factors are largely caused by relapse due to discontinuity or non-compliance to medication, unpleasant side effects and lack of insight [54, 57]. Accordingly, system-related factors are mostly associated to non-availability of medications, infrequent reviews by clinicians as well a switch of medications. Subsequently, the increasing negative perceptions about poor treatment outcomes have several implications on the ability of service users to continually use psychiatric services. Whilst there is an increasing negative perception of poor treatment outcomes, only a few studies have attempted to examine this from the service user and caregiver perspective and therefore underscore the need to understand the effectiveness as well as quality of mental health services.

As a common characteristic among LMICs, the negative perception about the poor quality of psychiatric medication outcomes usually influences service users to seek alternative care for such conditions. Subsequently, recognising the religious and cultural beliefs associated with mental illness demonstrates that service users are most likely to resort to faith-based healing as a treatment option.

## Implications for policy and future research

The study concludes that, although there appears to be increasing evidence regarding potential enablers and barriers to accessing mental health services, there is variation in the direction of these research results. In particular, the evidence on access to mental health services is skewed towards issues around attitudinal barriers, particularly stigmatising attitudes towards service user- and health system-related obstacles, with comparatively little or no evidence on the effectiveness or quality of mental health services provided to users.

Based on the findings from this integrative review, we can make several recommendations for policy and clinical practices, as follows:

- Advocacy for, and awareness about, existing mental health services should be funded and prioritised in policy initiatives.
- The existing psychiatric facilities should be resourced with infrastructure, modern equipment and logistics to effectively provide mental health services.
- The current funding provided by government to mental health services should be increased in order to cover regional and sub-regional level services.
- The existing task-shifting should be strengthened through efforts towards service integration and effective interprofessional training and supervision.
- An adequate supply of medication should be ensured through public spending and this should be supplemented by an exemption for service users in the existing National Health Insurance Scheme.

In addition, based on the current findings, some suggestions for future research are provided, as follows:

- Research on mental health services in Ghana should be directed towards measuring the outcome of services – effectiveness, efficacy, service user satisfaction and quality of life.
- The existing evidence has largely neglected the provision of mental health services for marginalised users, including children and young people, people with disabilities and the elderly. Future research should aim at ensuring adequate mental health service provision to these vulnerable populations.

### Additional file

Additional file 1: Methodological quality assessment criteria. (DOCX 22 kb)

## Appendix

**Table 4:** Data extraction form

Study ID	
Study details	
Citation	
Year of publication(s)	
Author(s)	
Contact details of lead author	
Funder/sponsoring organisation	
Publication type	
• Journal article • Report (specify) • Case study	
Other	
Publication source	
Methodology (if applicable)	
• Study design	
• Type of data	
• Data collection	
• Sampling	
• Data analysis	
• Participants/No. of studies included	
Population	
Mental illness	
• Please specify type of mental illness?	
Age range	
Sex	
Study setting	
Objective of the study	
Sector	
The paper may focus on one or more sectors, i.e.	
• Policy	
• Law	
• Data (prevalence, incidence)	
• Service delivery, i.e. formal, traditional	
• Enablers to care	
• Barriers to care	
• Effectiveness/impact of care	
• Other	
Policies and laws	Please specify the existing policies and laws on mental health services in Ghana?
Potential/effectiveness of policy	Please describe the potential of the existing policies on mental health?
Epidemiological and public health data on mental illness	Please specify the existing epidemiological and public health data on mental health in Ghana?
Mental health services	Please specify the extent of access and interventions that have been employed to improve access to mental health services for people with mental illness?
*Intervention*	
• Preferential treatment	
• Exemption to health insurance	
• Mental health drugs	
• Support care	
• Family support	
• Referral services	
• Training	
Other	
Please specify the following	
• Who implemented the intervention	
• Years of intervention	
Source of funding	
Potential/effectiveness of mental health services	Please describe the effectiveness of the existing mental health services/intervention?
Please describe barriers to timely access to mental health services?	
*Please be specific and concise*	
Please report any additional information on mental health services in Ghana	
Recommendation – methodological gaps and contribution to the specialty	
Identifiable references to follow-up	

## Abbreviations

CMHOs: Community mental health officers
CMHWs: Community mental health workers
CPNs: Community psychiatric nurses
CPOs: Clinical psychiatric officers
JBI: Joanna Briggs Institute
LMICs: Low- and middle-income countries
MNS: Mental, neurological and substance
PHC: Primary healthcare
