# Supplementary material for: An integrative review of potential enablers and barriers to accessing mental health services in Ghana
Source: Health Res Policy Syst. 2018 Nov 16;16:110. doi: 10.1186/s12961-018-0382-1 (PMC6240297; doi:10.1186/s12961-018-0382-1)
Supplement: Supplementary file 1 — Methodological quality assessment criteria. (DOCX 22 kb) [file 12961_2018_382_MOESM1_ESM.docx]

| **Reviewer 1** |  | | | |
| --- | --- | --- | --- | --- |
| **Reviewer 2** |  | | | |
| **Author (s)** |  | | | |
| **Methods** |  | | | |
| **Study design** |  | | | |
| **Data** |  | | | |
| **Sampling** |  | | | |
| **Analysis** |  | | | |
| **Types of Study** | **Methodological Quality assessment Criteria** | **Yes** | **No** | **Cant tell** |
| **Screening Questions (for all types)** | Are there clear research questions or objectives? |  |  |  |
|  | Do the collected data address the research question? |  |  |  |
|  | *Further appraisal is not feasible when the answer is ‘No’ or ‘Can’t tell’ to one or both screening questions* |  |  |  |
| **Qualitative** | 1.1 Is there congruity between the stated philosophical perspective and the research methodology? |  |  |  |
|  | 1.2 Are the sources of qualitative data (archives, documents, informants, observations) relevant to address the research question? |  |  |  |
|  | 1.3 Is the process for analysing qualitative data relevant to address the research question? |  |  |  |
|  | 1.4 Are participants, and their voices, adequately represented?  (adequate quotes and text been used to represent the concept discussed) |  |  |  |
|  | 1.5 Is there a statement locating the researcher culturally or theoretically? (Are the beliefs and values, and their potential influence on the study declared?) |  |  |  |
|  | 1.6. Is the influence of the researcher on the research, and vice- versa, addressed? *(Addressing the potential for the researcher to either influence or to be influenced by the study)* |  |  |  |
|  | 1.7. Do the conclusions drawn in the research report flow from the analysis, or interpretation, of the data? |  |  |  |
|  | 1.8. Is the ethical issues adequately addressed?  *(statement indicating appropriate ethics approval)* |  |  |  |
| **Quantitative**  **randomized controlled**  **(trials)** | 2.1. Is there a clear description of the randomization (or an appropriate sequence generation)? |  |  |  |
|  | 2.2. Is there a clear description of the allocation concealment or blinding when applicable)? |  |  |  |
|  | 2.3. Are there complete outcome data (80% or above)? |  |  |  |
|  | 2.4. Is there low withdrawal/drop-out (below 20%)? |  |  |  |
| **Quantitative non-**  **randomized**  **(Cohort study, case-control study, analytical cross-sectional)** | 3.1. Are participants recruited in a way that minimizes selection bias? |  |  |  |
|  | 3.2 Were the criteria for inclusion in the sample clearly defined? |  |  |  |
|  | 3.3 Were the study subjects and the setting described in detail? |  |  |  |
|  | 3.4 Were objective, standard criteria used for measurement of the condition? |  |  |  |
|  | 3.5 Were the outcomes measured in a valid and reliable way? |  |  |  |
|  | 3.6 Was appropriate statistical analysis used? |  |  |  |
|  | 3.7 Is the ethical issues adequately addressed?  (statement indicating appropriate ethics approval) |  |  |  |
|  | 3.8 Do the conclusions drawn in the research report flow from the analysis, or interpretation, of the data? |  |  |  |
|  | 3.9 Are measurements appropriate (clear origin, or validity known, or standard instrument; and absence of contamination between groups when appropriate) regarding the exposure/intervention and outcomes? |  |  |  |
|  | 3.11 In the groups being compared (exposed vs. non-exposed; with intervention vs. without; cases vs. controls), are the participants comparable, or do researchers take into account (control for) the difference between these groups? |  |  |  |
|  | 3.12 Are there complete outcome data (80% or above), and, when applicable, an acceptable response rate (60% or above), or an acceptable follow-up rate for cohort studies (depending on the duration of follow-up)? |  |  |  |
| **Quantitative**  **descriptive** | 4.1. Is the sampling strategy relevant to address the quantitative research question (quantitative aspect of the mixed methods question)? |  |  |  |
|  | 4.2. Is the sample representative of the population understudy? |  |  |  |
|  | 4.3. Are measurements appropriate (clear origin, or validity known, or standard instrument)? |  |  |  |
|  | 4.4. Is there an acceptable response rate (60% or above)? |  |  |  |
| **Systematic Review** | 5.1 Is the review question clearly and explicitly stated? |  |  |  |
|  | 5.2 Were the inclusion criteria appropriate for the review question? |  |  |  |
|  | 5.3 Was the search strategy appropriate? |  |  |  |
|  | 5.4 Were the sources and resources used to search for studies adequate? |  |  |  |
|  | Were the criteria for appraising studies appropriate? |  |  |  |
|  | 5.5 Was critical appraisal conducted by two or more reviewers independently? |  |  |  |
|  | 5.6 Were there methods to minimize errors in data extraction? |  |  |  |
|  | 5.7 Were the methods used to combine studies appropriate? |  |  |  |
|  | 5.8 Was the likelihood of publication bias assessed? |  |  |  |
|  | 5.9 Were recommendations for policy and/or practice supported by the reported data? |  |  |  |
|  | 5.10 Were the specific directives for new research appropriate? |  |  |  |
| **Mixed methods** | 6.1. Is the mixed methods research design relevant to address the qualitative and quantitative research questions, or the qualitative and quantitative aspects of the mixed methods question? |  |  |  |
|  | 6.2. Is the integration of qualitative and quantitative data (or results relevant to address the research question? |  |  |  |
|  | 6.3. Is appropriate consideration given to the limitations associated with this integration, e.g., the divergence of qualitative and quantitative data in a triangulation design? |  |  |  |
|  | *Apply the criteria use for qualitative data for the qualitative component and quantitative component respectively.* | | | |
| **Overall Quality Score** | *Comments on score:* | Low (25%)  Medium (50%)  High 75% - 100% | | |
| **Reviewer 2** | *Comments:* |  | | |
|  | *The score can be computed by counting the total number of “yes” and expressing them as a percentage ie below 25% represent Low Quality, 50% represent Medium Quality, and 75% and above represent high Quality.* |  | | |
